# 1601. Transmitted Drug Resistance in Treatment-Naïve People Living with HIV in Costa Rica

**DOI:** 10.1093/ofid/ofad500.1436

**Published:** 2023-11-27

**Authors:** Manuel Ramirez-Cardoce, Viviana Lopez-Bolanos

**Affiliations:** Hospital San Juan de Dios, CCSS, San Rafael, Alajuela, Costa Rica; Hospital San Juan de DIos, CCSS, San Jose, San Jose, Costa Rica

## Abstract

**Background:**

Transmitted drug resistance (TDR) occurs in people living with HIV infection (PLWH) who are not exposed to antiretroviral drugs but who are infected with a virus with mutations associated with resistance. In Costa Rica, HIV genotyping is performed in PLWH failing their first-line ART regimen, but it is not routinely done for all treatment-naïve PLWH before ART initiation, therefore, little is known about TDR prevalence.

**Methods:**

The aim of this study was to determine the prevalence of TDR and the HIV mutation patterns. During 2020, 162 cases of newly diagnosed treatment-naïve PLWH from all of the country’s HIV clinics were included for a genotyping study. Viral ARN was extracted from plasma samples obtained from the HIV National Reference Laboratory and protease (PR), reverse transcriptase (RT), and integrase (IN) regions were amplified and sequenced. The HIV database Program (Stanford University) and the World Health Organization (WHO) 2009 TDR surveillance mutation list were used for interpretation of resistance-associated mutations.

**Results:**

The overall prevalence of TDR was 36% (N=59/162). According to ART class, TDR was 22% in the nucleoside reverse transcriptase inhibitors (NRTI), followed by 13% in the non-nucleoside reverse transcriptase inhibitors (NNRTI); for the protease inhibitors (PI) and the integrase inhibitors (INI), TDR was 8% and 3%, respectively. (Figures 1-3). Most common mutations were: in NRTI, M41L (9.9%), and M184I (6.8%); in NNRTI, K103N (7.4%); in PI, M46I (4.3%); in INI, T97A (1.9%). (Table 1). The median age was 32 years-old (range: 16-71), and 91% were male. Median CD4 count was 420 cells/uL (range: 11-1670) and median viral load was 588337 copies/mL (range: 1220-10000000). According to CDC classification, 20% were stage C-3.

**Figure:**
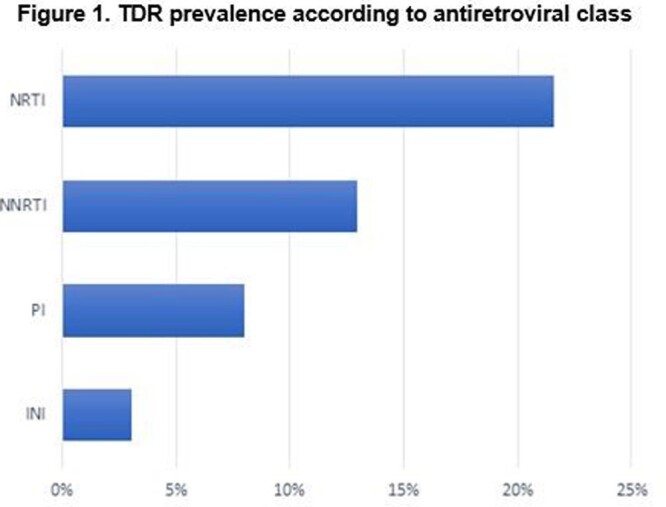

Source: local database

**Figure:**
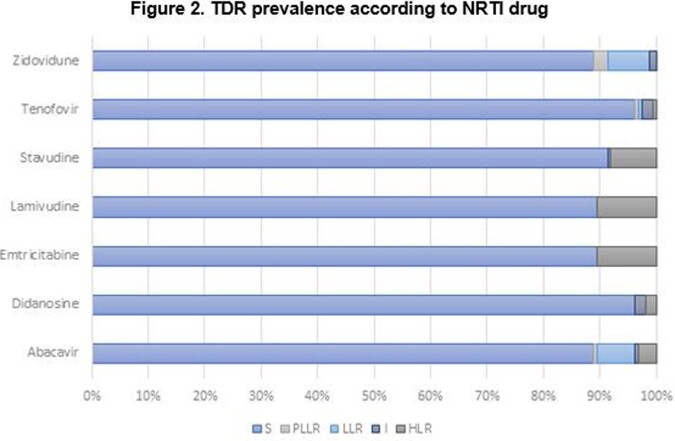

Source: local database

**Figure:**
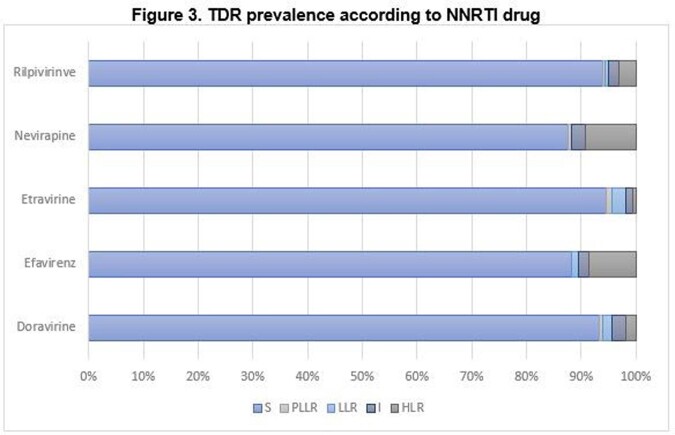

Source: local database

**Conclusion:**

This study population has a moderate level of HIV drug resistance (HIVDR) in ART-naïve PLWH and strongly implies the need to introduce HIVDR surveillance in Costa Rica. These findings confirm the TDR and highlight the need for systematic viral load monitoring and drug resistance testing at diagnosis. Continuous surveillance of newly infected individuals is required to help strategize the best ART regimen for these patients, and should prompt a change in current local recommendations for starting ART.

**Figure:**
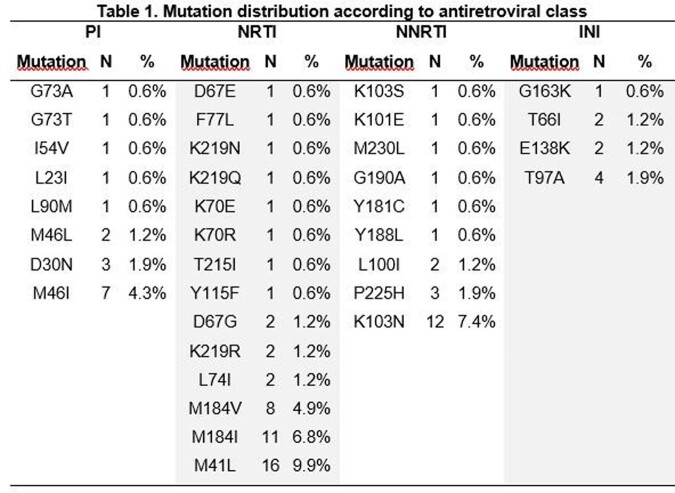

Source: local database

**Disclosures:**

**All Authors**: No reported disclosures

